# Signals of positive selection in mitochondrial protein‐coding genes of woolly mammoth: Adaptation to extreme environments?

**DOI:** 10.1002/ece3.5250

**Published:** 2019-05-09

**Authors:** Jacob Njaramba Ngatia, Tian Ming Lan, Thi Dao Dinh, Le Zhang, Ahmed Khalid Ahmed, Yan Chun Xu

**Affiliations:** ^1^ College of Wildlife Resources Northeast Forestry University Harbin China; ^2^ BGI‐Shenzhen Shenzhen China; ^3^ Laboratory of Genomics and Molecular Biomedicine, Department of Biology University of Copenhagen Copenhagen Denmark; ^4^ China National Genebank, BGI‐Shenzhen Shenzhen China; ^5^ State Forestry and Grassland Administration Research Center of Engineering Technology for Wildlife Conservation and Utilization Harbin China; ^6^ State Forestry and Grassland Administration Detecting Centre of Wildlife Harbin China

**Keywords:** *Mammuthus primigenius*, mitochondrial genome, mitolineages, positive selection, woolly mammoth

## Abstract

The mammoths originated in warm and equatorial Africa and later colonized cold and high‐latitude environments. Studies on nuclear genes suggest that woolly mammoth had evolved genetic variations involved in processes relevant to cold tolerance, including lipid metabolism and thermogenesis, and adaptation to extremely varied light and darkness cycles. The mitochondria is a major regulator of cellular energy metabolism, thus the mitogenome of mammoths may also exhibit adaptive evolution. However, little is yet known in this regard. In this study, we analyzed mitochondrial protein‐coding genes (MPCGs) sequences of 75 broadly distributed woolly mammoths (*Mammuthus primigenius*) to test for signatures of positive selection. Results showed that a total of eleven amino acid sites in six genes, namely ND1, ND4, ND5, ND6, CYTB, and ATP6, displayed strong evidence of positive selection. Two sites were located in close proximity to proton‐translocation channels in mitochondrial complex I. Biochemical and homology protein structure modeling analyses demonstrated that five amino acid substitutions in ND1, ND5, and ND6 might have influenced the performance of protein–protein interaction among subunits of complex I, and three substitutions in CYTB and ATP6 might have influenced the performance of metabolic regulatory chain. These findings suggest metabolic adaptations in the mitogenome of woolly mammoths in relation to extreme environments and provide a basis for further tests on the significance of the variations on other systems.

## 1 INTRODUCTION

The genera *mammuthus* is believed to have originated in warm and equatorial Africa around 6.7–7.6 million years ago (Rohland et al., 2007) and later colonized cold and high‐latitude environments in the arctic (Lister & Bahn, 2007). The woolly mammoth, *Mammuthus primigenius*, was the last living species of this genera, whose final population on Wrangel Island survived until about 4,000 years ago (Palkopoulou et al., 2015). Genomic studies have suggested three major clades in woolly mammoths (Chang et al., 2017; Palkopoulou et al., 2013). Clade 1 includes three mitochondrial haplogroups, namely C, D, and E, which occurred in North America and Eurasia. Clade 2/A occurred in Asia, but was geographically restricted to northern Siberia and western Beringia, where it coexisted in sympatry with Clade 1(haplogroups D and E) until its extinction (Barnes et al., 2007; Chang et al., 2017; Debruyne et al., 2008). Clade 3 which includes haplogroups B1 and B2 were found in Alaska/Yukon of North America and Europe, respectively (Chang et al., 2017; Palkopoulou et al., 2013).

The woolly mammoths were suggested to have lived in high‐latitude environments with extreme climatic (cold) and dry conditions (MacDonald et al., 2012). Studies have suggested that nuclear genes of woolly mammoth had evolved genetic variations involved in processes relevant to cold tolerance, including lipid metabolism and thermogenesis, and adaptation to extremely varied light and darkness cycles (Smith, Kawash, Karaiskos, Biluck, & Grigoriev, 2017). However, the most essential role in generating energy in eukaryotic cells is played by the mitochondria where up to 95 percent of energy is generated through oxidative phosphorylation (OXPHOS) system (Levin, Blumberg, Barshad, & Mishmar, 2014). Different mitochondrial variants may have different effects on cellular and organismal metabolic performance (Ballard & Melvin, 2010). Thus, it could be expected that the mitochondrial genome of the woolly mammoth would have evolved in association with nuclear genome in adaptation to such extreme environments. Up to the present, little is known regarding adaptive evolution of their mitochondrial genes.

In vertebrates, 13 mitochondrial protein‐coding genes (MPCGs) are involved in phosphorylation process, which includes seven subunits of NADH dehydrogenase (ND), one subunit of cytochrome *b* (cyt *b*), two subunits of ATP synthase (ATP), and three subunits of cytochrome c oxidase (COX) (Wallace, 2007). These subunits are fundamental in electron and proton transportation, and respiratory regulation of the mitochondria (Bai, Shakeley, & Attardi, 2000). In addition, the subunits have close association with those encoded by nuclear genome in the OXPHOS pathway (Rand, Haney, & Fry, 2004). Thus, OXPHOS functions can be influenced by mutations occurring in either genome, and mitogenome products are especially important to directly mediate proton pumping across the mitochondrial membrane (Gu et al., 2016). These findings suggest that co‐evolution between the two genomes has been favored by natural selection to promote optimal maintenance of metabolic functions.

The impact of any amino acid substitution can differ significantly depending on its position in the protein structure. Therefore, site‐specific mutations that do not indicate changes in protein structure can influence function, for example changes in the binding sites. Amino acid substitutions in the mitochondrial subunits can influence the efficiency in the electron transport chain system, that can hinder or improve the OXPHOS process (Fonseca, Johnson, O'Brien, Ramos, & Antunes, 2008) and can be linked to environmental adaptations (Almeida, Maldonado, Vasconcelos, & Antunes, 2015). Adaptive changes observed in the MPCGs have been associated with environmental conditions, such as latitudinal clines (Stager, Cerasale, Dor, Winkler, & Cheviron, 2014), anoxic conditions (Tomasco & Lessa, 2011), and altitude (Yu, Wang, Ting, & Zhang, 2011).

Here, we compared comprehensive dataset of mitogenome sequences of woolly mammoth's to identify positively selected sites in the MPCGs, assessed the biochemical changes in amino acids and applied structure‐based homology comparison models to predict the effect of these changes in the protein structure and functioning of the OXPHOS pathway.

## 2 METHODS

### 2.1 Sequence collection

**Figure 1:**
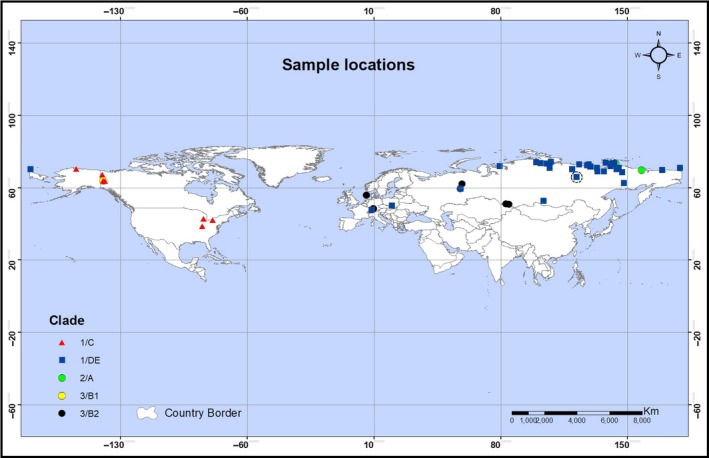
Sites locations of woolly mammoth mitochondrial genome samples included in this study. The samples are color‐coded by clade. The location of the five samples we collected and used for this study is shown as a blue square enclosed with a dotted circle, of which the latter represents the approximate diameter for their geographic range

Unpublished whole genome sequence data of six woolly mammoth (*Mammuthus primigenius*) samples were downloaded from NCBI GenBank (https://www.ncbi.nlm.nih.gov: Accession number ERP111819) or CNSA (https://db.cngb.org/cnsa: Accession number CNP0000209), and subsequently used to generate complete mitochondrial genomes. Raw reads were filtered using AdapterRemoval (Lindgreen, 2012) with the minimum base quality set to 15 and the minimum read lengths set to 30. The first 2 bases and the last 3 bases at the ends of each read were trimmed to prevent DNA damage‐induced errors because deamination is more likely to occur at the ends of ancient DNA fragments. The C‐to‐T and G‐to‐A changes that occurred at the first 10 and last 10 bases of each read were also removed to further avoid potentially incorrect bases. Filtered reads were aligned to the reference genome of African elephant (loxAfr4, ftp://ftp.broadinstitute.org/distribution/assemblies/mammals/elephant/loxAfr4/) with the mitochondrial genome being replaced by woolly mammoth mitogenome (Acc No. DQ188829.2) using BWA (Li & Durbin, 2009) with the *aln* mode using parameters: ‐l 30 –k 2 –t 4. The multi‐mapped reads were removed to obtain uniquely mapped reads. SAMtools (Li et al., 2009) was then used to remove the unmapped reads as well as reads with a mapping quality less than 1. We finally drew a random sequence for each site on the reference mitogenome to retrieve the mitochondrial genome for each of the six samples (Yang et al., 2017). The retrieved mitochondrial genome sequence of one of the six samples (N6) was found to contain numerous sites with gaps. Thus, it was not included in further analyses. Seventy additional mitogenome sequences (Chang et al., 2017; Enk et al., 2016; Fellows Yates et al., 2017; Gilbert et al., 2008, 2007; Krause et al., 2006; Rogaev et al., 2006) were downloaded from GenBank and included in the analysis (Table S1). These samples were chosen based on the previously published mitolineage (clade) information, geographic origin, and the completeness of the genome sequences. The selected mitogenome sequences were complete or with relatively fewer gaps in the mitochondrial protein‐coding genes region and were considered more suitable for subsequent analyses. The geographic origins of the 75 samples are shown in Figure 1. Previously published mitogenome sequences of *Mammuthus columbi* (GenBank accession No JF912199) and *Mammuthus jefersonii* (GenBank accession No KX027559) were also included in this study.

### 2.2 Alignment and phylogenetic analyses

Thirteen protein‐coding gene (ND1‐ 6, ND4L, ATP6, ATP8, COX1, COXII, COXIII, CYTB) sequences were extracted from complete mitogenome and concatenated using Geneious Pro 7.0.6 (Kearse et al., 2012). Nucleotide sequences were aligned using ClustalW in MEGA 5 (Tamura et al., 2011), where the Asian elephant (GenBank accession No EF588275) was used as an outgroup. Alignments and subsequent analyses including positive selection were performed independently for each gene. But for phylogenetic analyses, concatenated sequences of the coding genes were considered. Here, Bayesian analysis (MB) was done using MrBayes (Ronquist & Huelsenbeck, 2003). The best‐fit model of evolution was identified using jModelTest (Darriba, Taboada, Doallo, & Posada, 2012), through the Akaike information criterion (AIC) (Bozdogan, 1987). But because the selected model (TVM + G) is not implemented by MrBayes, it was substituted by GTR model (Lecocq et al., 2013). Bayesian MCMC (Markov chain Monte Carlo) was run for 2 million generations and sampling every 1,000 generations. The phylogenetic tree was summarized in MrBayes after removing the first 25% of the tree as burn‐in. The consensus tree was then visualized using Fig Tree. With respect to selection analyses, nucleotide sequences for each gene were separately aligned against the reference sequence Acc. No EF588275. Maximum likelihood (ML) trees included in TreeSAAP analysis were constructed using models of evolution identified as mentioned above (Table 1).

**Table 1:** The selected models of evolution for each protein‐coding gene as inferred by jModelTest

Gene	Model
ND1	TPm3uf + I
ND2	TIM3 + I
ND3	HKY + G
ND4L	TPM2uf
ND4	HKY + I
ND5	HKY
ND6	HKY + I
CYTB	HKY + G
COX I	TIM2 + G
COX II	TrN
COX III	TPM1uf + I
ATP6	TPM3uf
ATP8	HKY

### 2.3 Selection analyses

The widely used method for detecting selection in protein‐coding genes is by estimating ω, the nonsynonymous/synonymous substitution rates ratio (*d*_N_/*d*_S_ *)* (Yang, 1998). However, this approach for identifying genetic adaptation is mostly biased against even the proteins that are moderately conservative, since it does not accommodate the likelihood that adaptation may result from a few amino acid changes. Therefore, significant changes in physicochemical properties among protein residues in a phylogenetic tree were detected using TreeSAAP, which compares observed distribution of physicochemical changes deduced from a phylogenetic tree with expected distributions based on an assumption that completely random amino acid substitutions are expected under selective neutrality conditions (Woolley, Johnson, Smith, Crandall, & McClellan, 2003). To reduce false positives in TreeSAAP analysis, we included only 20 amino acid properties showing >85% accuracy of identifying positive selection, set a sliding window equal to 15 codons, and only considered amino acid changes with radical effects on physicochemical properties (magnitude categories ≥ 6 and *p* < 0.001). Significant positive *Z*‐scores imply that higher magnitude nonsynonymous mutations are more frequent than expected under neutrality, suggesting change due to positive selection (McClellan & Ellison, 2010; Woolley et al., 2003). Significant nonsynonymous mutations found to occur at the clade or haplogroup level were considered as candidates of positive selection in this study.

### 2.4 Protein modeling and structural analyses

In order to test the effect of the biochemical changes of the selected sites on the protein structure of *M. primigenius*, the protein sequences of each subunit of complex I were aligned with sequences of homologous subunits in GenBank (https://www.ncbi.nlm.nih.gov/) protein database. Protein homology models of individual subunits were then predicted using the automated mode program (http://swissmodel.expasy.org/workspace/index) of the SWISS‐MODEL server using the best hit templates. The best hit homology templates for each subunit were as follows: ND1 (PDB: 5lnk.1.H) and ND5 (PDB: 5lnk.1.M), both from *Ovis aries*: ND2 (PDB: 5gup.34.A), ND3 (PDB: 35gup.35.A), ND4L (PDB: 5gup.36.A), and ND4 (PDB: 5gup.42.A) that were all from *Sus scrofa*: and ND6 (PDB: 5lc5.1.J) from *Bos Taurus*. An overall structure of the *M. primigenius* complex I was then generated by structurally aligning individual subunits to corresponding complex I template from *Ovis aries* (PDB:5lnk) using Chimera software ( Pettersen et al., 2004). The modeled complex I structure was refined by adjusting the side chains using FoldX software (Schymkowitz et al., 2005). After modeling complex I structure, solvent accessibility area (ASA) of each residue under positive selection was computed using GETAREA program (Fraczkiewicz & Braun, 1998) and determined whether they were located on protein binding sites using PRODIGY (Vangone & Bonvin, 2015; Xue, Rodrigues, Kastritis, Bonvin, & Vangone, 2016). Residue locations on the protein were established using TMHMH v2.0 (2001). Intraprotein cavities and pathways were examined with program CAVER (Petřek et al., 2006). Functionally important residues were determined using ConSurf (Glaser et al., 2003). Graphic modifications and presentations were finally depicted using PyMOL (DeLano, 2002).

## 3 RESULTS

### 3.1 Phylogenetic analyses

**Figure 2:**
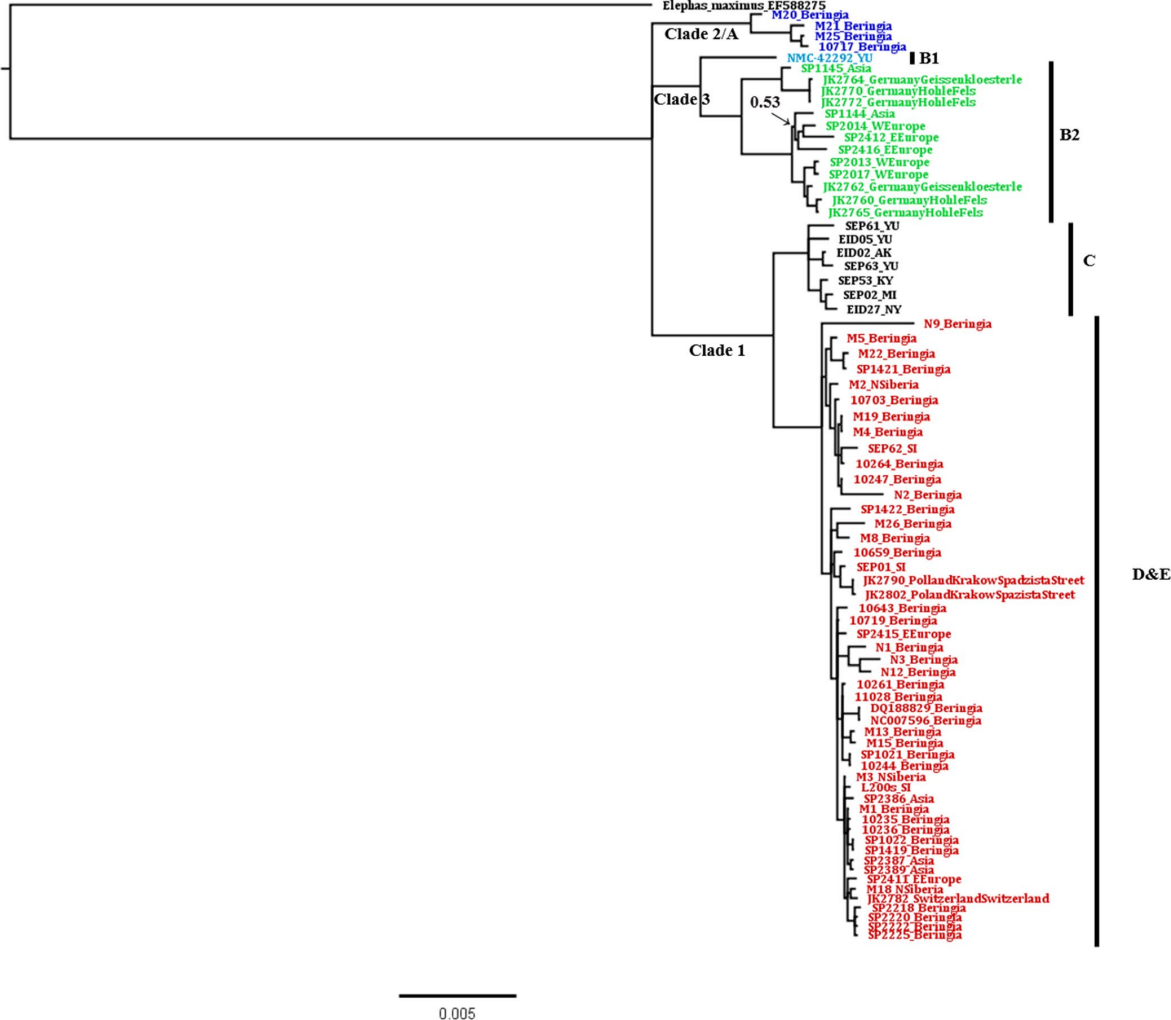
Rooted phylogeny of *Mammuthus primigenius* based on mtDNA protein‐coding sequences estimated by Bayesian inference. All the nodes are well supported (PP > 0.9) unless indicated otherwise; mtDNA clades and the respective haplogroups labeled on the tree branches and on the right side of the tree respectively; the taxa names and codes are the individual sequence ID codes

Based on the concatenated sequences of 13 protein‐coding genes (11,412 bp), a phylogenetic tree constructed using MrBayes showed distinct woolly mammoth's mitolineages and was congruent with those previously constructed using complete mitochondrial genomes (Chang et al., 2017; Gilbert et al., 2008). Most nodes were well supported (posterior probability (PP) > 0.9), with each clade showing PP of 1. All the sequences prepared in this study (N1, N2, N3, N9, and N12) were found to belong to Clade 1 haplogroups D and E (Figure 2).

### 3.2 Evidence of positive selection in mitochondrial genes

TreeSAAP, which is highly sensitive to even the most conservative proteins, detected sixteen significant (*p* < 0.001) and radical (categories 6–8) physiochemical changes in amino acid properties in eight of the thirteen protein‐coding genes (Table 2). The nonsynonymous mutations identified in codons ND1, ND2, ND4, ND6, Cyt *b*, ATP6, and COX1 showed significant changes in equilibrium constant (ionization of COOH) (*pK′*). In ND5, the mutation influenced radical changes in average number of surrounding residues (*N*_s_), equilibrium constant (ionization of COOH) (*pK′*), buriedness (Br), solvent accessible reduction ratio (Ra), surrounding hydrophobicity (Hp), and thermodynamic transfer hydrophobicity (Ht) (Table 2). Changes in *pK′* imply changes in ionization properties of the amino acids while changes in *N*_s_, Br, Ra, Hp, and Ht indicate changes in hydrophilicity or compactness in the surrounding region of the mutated residue (Cozzone, 2010).

**Table 2:** Nonsynonymous substitutions influencing changes in biochemical properties as inferred by TreeSAAP

Gene (codon)	Codon changes	Amino acid replacement	Physicochemical properties					
			*N*_s_	*pK*′	Br	Ra	Hp	Ht
ND1(6)	ATC‐GTC	Ile‐Val		↑8				
ND1(73)	ATT‐GTT	Ile‐Val		↑8				
ND2 (282)	ATG‐ATA	Met‐ Ile		↓8				
ND4 (87)	GAA‐GGA	Glu‐Gly		↓8				
ND5 (16)	ATC‐ACC	Ile‐Thr	↓6	↑6	↓6	↓7	↓6	↓7
ND5 (20)	ACC‐ATC	Thr‐Ile	↑6	↓6	↑6	↑7	↑6	↑7
ND5 (51)	ATT‐ACT	Ile‐Thr	↓6	↑6	↓6	↓7	↓6	↓7
ND5 (186)	ATG‐ATA	Met‐Ile		↓8				
ND5 (191)	ACC‐ATC	Thr‐Ile	↑6	↓6	↑6	↑7	↑6	↑7
ND5 (512)	ACA‐ATA	Thr‐Ile	↑6	↑6	↑6	↑7	↑6	↑7
ND6 (4)	ATT‐GTT	Ile‐Val		↑8				
CytB (189)	ATG‐ATA	Met‐Ile		↓8				
CytB (300)	GTC‐ATC	Val‐Ile		↓8				
CytB (371)	ATA‐ATG	Ile‐Met		↑8				
ATP6 (110)	GTC‐ATC	Val‐Ile		↓8				
COX1(190)	GTT‐ATT	Val‐Ile		↓8				

Note

The codon number corresponds to the coding alignment of each gene as applied in this study. The values in the physicochemical properties columns represent the category of radical change in each property, where (↑) shows an increase in magnitude, while (↓) shows a decrease. Amino acid change was significant at categories 6–8.

*N*_s_ = number of surrounding residues; *pK*′ = equilibrium constant (ionization of COOH);* Br* = buriedness; Ra* = *solvent accessible reduction ratio; Hp* = *surrounding hydrophobicity; Ht = thermodynamic transfer hydrophobicity.

Nonsynonymous mutations detected herein mostly influenced changes in amino acids ionization properties (*Pk*′) (Table 2). Changes in ionization properties of the amino acids, including those of their side chains significantly influence protein's stability, solubility, and structural organization. At low ionic strength, most protein's solubility is relatively high, but it reduces with increase in the ionic strength (Cozzone, 2010). Similarly, the hydrophilicity/hydrophobicity of the side chains influences the physicochemical behavior of polypeptide chains including their foldability into three‐dimensional structures (Cozzone, 2010). Therefore, changes in physicochemical properties in amino acids may have possibly affected the protein tertiary structures. This question was resolved by locating these residues in the protein structures.

### 3.3 Amino acid substitutions in complex I

**Figure 3:**
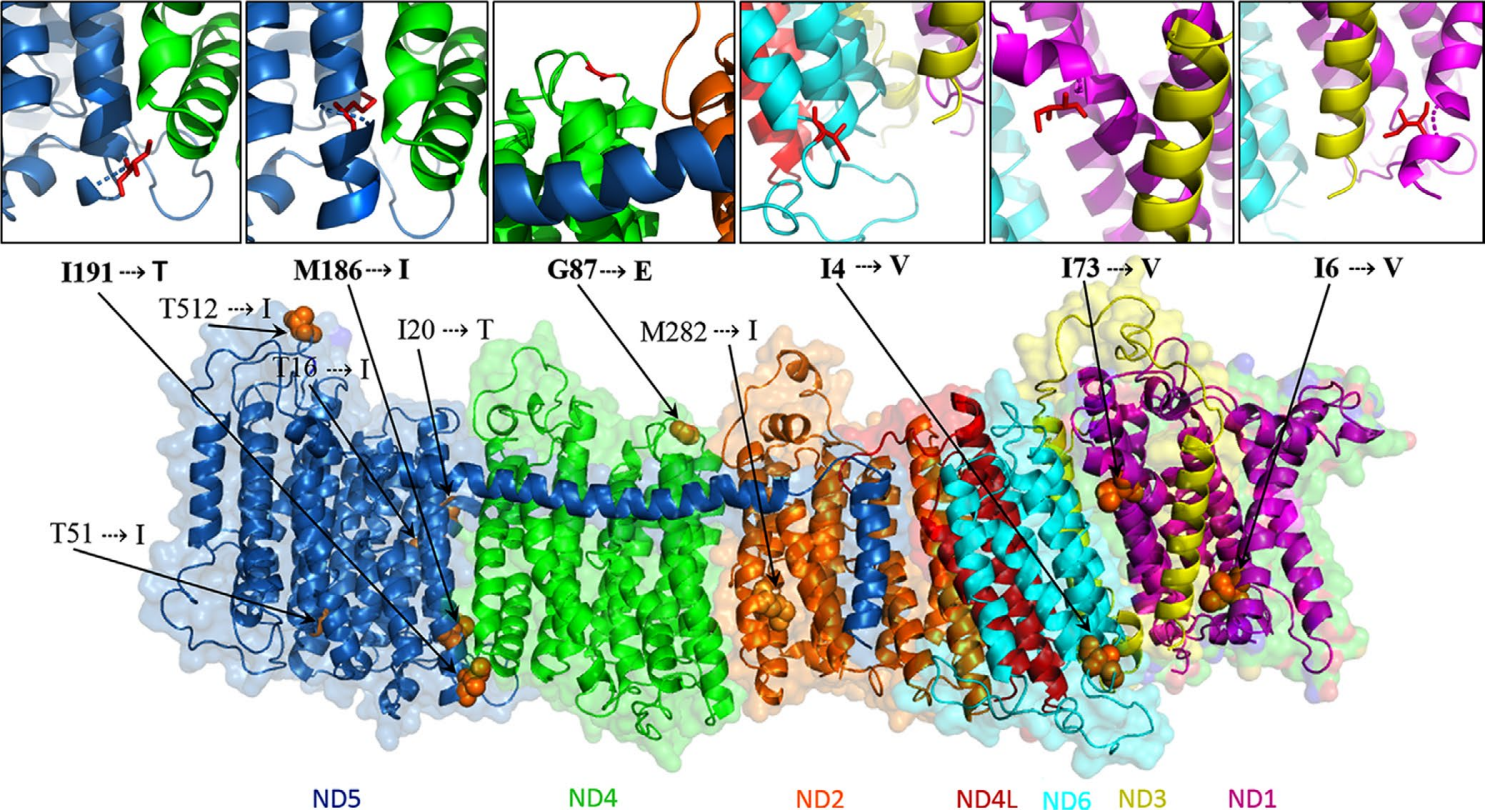
Atomic level structure of the predicted mitochondrial complex I of *M. primigenius*. Residues with amino acid substitutions are displayed as orange spheres. The potentially active sites are enlarged images shown as red sticks. Letters before the residues indicate the amino acids and their corresponding substitutions

The homology protein structures of six out of seven OXPHOS complex I protein chains showed high % similarity (>63%) to the parent structure, suggesting a fair degree of structural similarities (Table 3). After homology modeling and side chain refinement of complex I structure, free loops that did not align with *O. aries* structure were excluded resulting to a final complex I structure of the woolly mammoth (Figure 3).

**Table 3:** Calculations of protein structures prediction

Complex	Gene	Parent template	Species	% Similarity to Parent structure	Residues predicted	Reference
I	ND1	5lnk.1.H	*Ovis aries*	78.55	1–317	Fiedorczuk et al. (2016)
I	ND2	5gup.34.A	*Sus scrofa*	63.11	1–347	Wu, Gu, Guo, Huang, and Yang (2016)
I	ND3	5gup.35.A	*Sus scrofa*	78.95	1–114	Wu et al. (2016)
I	ND4L	5gup.36.A	*Sus scrofa*	73.47	1–97	Wu et al. (2016)
I	ND4	5gup.42.A	*Sus scrofa*	74.2	1–438	Wu et al. (2016)
I	ND5	5lnk.1.M	*Ovis aries*	69.97	1–597	Fiedorczuk et al. (2016)
I	ND6	5lc5.1.J	*Bos taurus*	57.89	1–171	Zhu, Vinothkumar, and Hirst (2016)

Note

The parent template and species columns show the best hit parent structures used in predicting the individual protein chains, and the corresponding species, respectively.

All the positively selected sites identified in complex I were mapped onto their corresponding subunits (Figure 3), to evaluate their locations and their relevance in protein structure and function. These sites were found in locations suggested not to be greatly conserved across species (Fiedorczuk et al., 2016). Mutations in five out of eleven residues in complex I were found in protein–protein binding sites where they interact with residues encoded by other subunits of complex I (Table 4). Amino acid changes at binding sites can influence binding properties of a residue including binding affinity, specificity, and foldability, which may affect protein–protein interaction during complex assembly and translocation process (Brender & Zhang, 2015). In addition, one substitution involved unrelated amino acids, which could likely cause changes in protein functions.

**Table 4:** Residue interactions with other protein subunits of complex I based on predictions from the protein structure

Gene (residue)	TM	Total TM	Binding residue	Interacting subunit (residues)	Relative accessible surface area (%)
ND1 (6)	1	8	+	ND3 (6,10,2,5,9)	0.1
ND1 (73)	2		+	ND6 (32)	32.7
ND2 (282)	7	8	−	—	0.0
ND4 (87)	Inside	12	−	—	0.0
ND5 (16)	1	14	−	—	50.9
ND5 (20)	1		−	—	38.6
ND5 (51)	2		−	—	15.6
ND5 (186)	5		+	ND4 (379)	0.5
ND5 (191)	Inside		+	ND2 (383,386,387,390)	27.5
ND5 (512)	Inside		−	—	66
ND6 (4)	1	5	+	1 (ND3)	54.5

Note

Inside indicates that the residue was located in the cytoplasmic matrix. TM represents the transmembrane.

At residue ND1_6_, isoleucine was substituted with valine. Here, all Clade I (haplogroups C, D, and E) and Clade 3 (haplogroups B1 & B2) along with M20 of Clade 2/A shared valine, while the rest of Clade 2/A shared isoleucine (Table 5). Chain ND1 is comprised of 8 transmembrane (TM) helices. Mutation in ND1_6 _was found in TM1. ND1_6 _was predicted to be a buried residue located in a protein–protein binding site, where it interacts with five other residues of subunit ND3. Both isoleucine and valine are aliphatic and hydrophobic, but valine is small (Taylor, 1986). Isoleucine and valine are not that dissimilar in their biochemistry, therefore mutation in residue ND1_6_ is not expected to cause direct changes in protein functions (Betts & Russell, 2003). But considering its location in an active binding site where it interacts with residues encoded by ND3 subunit, mutations at this site may affect the recognition of hydrophobic ligands (Betts & Russell, 2003) and protein–protein interactions during the assembly of OXPHOS complex I.

**Table 5:** Amino acid differences on positively selected codons among the woolly mammoth mitolineages as inferred by TreeSAAP

Gene	Codon No.	*E. maximus*	*M. columbi*	*M. jefersonii*	*M. primigenius*				
					Clade I		Clade 2	Clade 3	
					C	D & E	A	B1	B2
ND1	6	V	V	V	V	V	I^(3)^‐V^(1)^	V	V
ND1	73	V	V	V	V	V^(48)^‐I^(2)^	I	V	V
ND2	282	M	I	I	I	I	M	M	M
ND4	87	G	G	G	G	G	G	E	E
ND5	16	T	T	T	T	T	I	T	T
ND5	20	T	T	T	I	T	T	I	I
ND5	51	T	T	T	T	T	I	T	T
ND5	186	I	I	I	I	I	M	I	I
ND5	191	I	I	I	T	T	I	I	I
ND5	512	T	I	I	I	I	T	T	T
ND6	4	I	I	I	I	I	V^(3)^‐I^(1)^	I	I
Cyt *b*	189	I	I	I	I	I	I	M	M
Cyt *b*	300	I	I	I	I	I	I	V	V
Cyt *b*	371	I	M	M	M	M	I	I	I
ATP6	110	V	I	I	I	I	V	V	V
COX1	190	V	V	V	I	V	V	V	V

Note

The genus *M* represents *Mammuthus*. Numbers in the parenthesis indicate the number of individuals containing corresponding amino acids at each site.

Abbreviations: E, Glutamate; G, Glycine; I, Isoleucine; M, Methionine; T, Threonine; V, Valine.

At residue ND1_73_, substitution of isoleucine for valine was located in TM2. Here, all Clade 1 and Clade 3 shared valine except for samples NC007596 and DQ188829, both from Beringia which displayed isoleucine along with Clade 2/A. ND1_73 _was predicted to be located near a possible proton‐translocation pathway and binding site, where it interacts with a residue on the chain encoded by subunit ND6 (Table 4). In addition, ND1_73 _interacts with residue ND6_30 _that is near a functional residue ND6_32_ suggested to be important in proton translocation (Sazanov, 2015). As such, mutations at this site could possibly affect the functional state of the proton‐translocation pathway by coordinating the interacting residues.

Mutations in ND4_87_ (glutamate to glycine) were located at the loop region and within the cytoplasmic matrix. This site is near the proposed proton‐translocation pathway (Fiedorczuk et al., 2016; Sazanov, 2015). Here, all Clade 1 and Clade 2/A displayed glutamate, while Clade 3 showed glycine. Glutamate is charged and polar, while glycine is hydrophobic and small. Glutamate can be involved in salt bridges and play a role in catalytic sites of proteins (Betts & Russell, 2003), whereas glycine can play a functional role by binding to phosphates of the ATP molecule (Schulze‐Gahmen, Bondt, & Kim, 1996). Considering the location and physicochemical dissimilarities of amino acids change at ND4_87_, mutation at this site might have caused possible changes in the proton‐translocation channel and influenced its efficiency.

At ND5, which contains fourteen TM Helices, amino acid replacements observed at ND5_16 _(isoleucine to threonine) and ND_20_ (threonine to isoleucine), were both located at TM1. Residue ND5_16_ was a surface residue, while ND5_20_ was near the surface. All Clade 1 shared threonine at ND5_16_, while Clade 2/A had isoleucine. On the other hand, isoleucine at ND_20 _was shared by all Clade 1 while threonine was present in Clade 2/A. Isoleucine is hydrophobic and aliphatic, with threonine being hydrophobic, polar, and small. Unlike isoleucine, threonine contains a fairly reactive hydroxyl group, which can form hydrogen bonds with various substrates (Betts & Russell, 2003). These two residues (ND5_16_, ND5_20_) were not located in binding sites, but considering their location on/near the surface, the nature of amino acid substitution, and the role of the mutations in driving radical changes of six physicochemical properties, it is highly likely that they had a functional role.

Mutations in residues ND5_186_ and ND5_191_ were both located at protein–protein binding sites, which interacts with one residue on chain encoded by ND4, and four residues of chain encoded by ND2 subunits, respectively (Table 5). Subunits ND2, ND4, and ND5 are suggested to be proton‐pumping machines that are related to the Na^+^/H^+^ antiporters of the multiple resistance and PH (Mrp) family (Fonseca et al., 2008). Interestingly, substitution in ND5_186_ and ND5_191_ were located near a possible Na^+^ translocation channel, which has been proposed in *Escherichia coli* complex I (Sazanov, 2015). Previous evidence suggests that Na^+^ could be playing a direct role in catalytic mechanisms of complex 1 (Batista, Marreiros, & Pereira, 2012). Specifically, site ND5_186_ was predicted to be a buried residue located in the TM5. Here, all Clade 1 and Clade 3 shared isoleucine while Clade 2/A displayed methionine. Both methionine and isoleucine are hydrophobic, but isoleucine is aliphatic (Betts & Russell, 2003; Taylor, 1986). Meanwhile, the mutation in ND5_191_ was located in cytoplasmic region, where threonine was replaced with isoleucine. At this site, all Clade 1 had threonine, while Clade 2/A and Clade 3 shared isoleucine (Table 5). Taking into consideration that ND5_186_ and ND5_191_ are binding sites for other residues in chains encoded by ND4 and ND2 subunits, respectively, coupled with their location near a possible Na^+^ translocation channel, mutations at these residues might have likely influenced protein functions including catalytic mechanisms in complex I. Mutations affecting H^+^/ Na^+ ^transport activity has been found in dolphins, although their key role in adaptive selection could not be determined (Caballero, Duchêne, Garavito, Slikas, & Baker, 2015). Lastly, substitution ND6_4_ (isoleucine for valine) was located in TM1. ND6 has 5 TM's in total. At ND6_4_, which is located at the loop region, all Clade 1, Clade 3, and M20 of Clade 2/A displayed isoleucine, while the rest of Clade 2/A shared valine (Table 5). This substitution was found at the surface and binding site for a functional residue (site 1, at the N‐terminus of ND3), which makes it more likely to influence the overall protein structure and efficiency of the proton‐translocation channel which ND6 forms with ND1 and ND4L subunits.

The amino acids substitutions identified above are not that dissimilar in biochemistry, but considering their location, they may likely drive conformational changes in the proteins and the efficiency of the translocation processes including OXPHOS process.

### 3.4 Amino acid substitutions of complex III, IV, and V

We also observed significant amino acid substitutions in cyt *b*, COX1, and ATP6 subunits, which are membrane‐bound proteins found in mitochondrial complex III, IV, and V, respectively. Due to the difficulty in crystalizing membrane‐bound proteins, there is still limited structural information about the subunits such as for F0 proton channel in ATP6 important in OXPHOS process (Weber & Senior, 1997) and the hemes' dynamics in complex III which are important in electron transport chain (Kim, Khalimonchuk, Smith, & Winge, 2012). Therefore, we did not perform additional structural analysis of the aforementioned protein chains, but rather examined their biochemical differences.

Amino acid replacements in cyt *b* were found at residues 189, 300, and 371. At site cyt *b*_189_, methionine was substituted with isoleucine, where isoleucine was shared by Clade 1 and 2/A, while methionine was private to Clade 3 (Table 5). Both methionine and isoleucine are hydrophobic, but isoleucine is aliphatic. In residue cyt *b*_300,_ valine was substituted with isoleucine. Here, all Clade 1 and Clade 2/A shared isoleucine while Clade 3 haplogroups displayed valine. Both of these amino acids are hydrophobic but valine is small. At cyt *b*_371_, isoleucine was replaced by methionine, whose properties have been mentioned above. At this site, Clade 1 displayed methionine while Clade 2/A and Clade 3 shared isoleucine. With respect to COX1, mutations were found at site 190. Here, valine was replaced with isoleucine, where all Clade 1 shared isoleucine, while the rest of the clades shared valine (Table 4). Again, both of these amino acids are hydrophobic with valine being small. Lastly, at ATP6_110_, valine was substituted with isoleucine. At this site, all Clade 1 shared isoleucine while the rest of the clades shared valine. Generally, mutations in the cyt *b*, COX1, and ATP6 residues involved closely related amino acid residues, thus, they were not expected to drive significant changes in protein structure and function ( Betts & Russell, 2003).

## 4 DISCUSSION

Woolly mammoth comprised of endemic mitolineages that is Clade 2/A and Clade 1 haplogroup C that were exclusively Asian and North American in distribution, respectively, whereas, Clade 1 (haplogroups D & E) and Clade 3 were predominantly distributed in Asia and Europe, respectively. Such geographic pattern suggests a high level of intercontinental differentiation (Debruyne et al., 2008) and survival in varied environments. In order to detect positive selection in the mitogenome in woolly mammoths, physicochemical changes among codons in a phylogenetic tree were analyzed coupled with inferences of the potential effects of amino acid substitutions on the protein structure and function.

At the biochemical level, we found sixteen sites with strong positive selection leading to changes in amino acids physicochemical properties in ND1, ND2, ND4, ND5, ND6, CYTB, ATP6, and COX1 (Table 2). All the thirteen observed amino acid substitutions in the aforementioned genes affected the ionization properties of the amino acids, while five of them also led to changes in five other properties influencing hydrophilicity or compactness in the region surrounding the residues under mutation. Changes in these functional properties may compromise protein's stability, solubility, structural organization, and foldability. These findings showed slight disparity with the previous studies in woolly mammoths, where positive selection signal in complex I genes was not detected (Gilbert et al., 2008; Pečnerová et al., 2017), possibly because we used expanded dataset for divergent clades with a larger geographic coverage, thus providing a larger number of variable codons. We also drew inferences from TreeSAAP algorithm that is highly sensitive to even the most conservative proteins.

Our findings suggest that positive selection might have had a preferential effect on residues in NADH dehydrogenase (ND) complex (complex I). The residue mutations in complex I subunits were found to occur mainly in the transmembrane helices and regions that are not greatly conserved (Fiedorczuk et al., 2016). With respect to ND1 subunit, mutations were found in residues located at protein–protein binding sites. ND1 is fundamental in the assembly of complex I, since they serve as electron transport conduits, from ubiquinone to the binding pocket in the Q‐Module, thus, changes in ND1 can affect the assembly of the entire complex (Cardol, Matagne, & Remacle, 2002). Moreover, mutations in binding sites may affect protein–protein interaction during complex assembly and translocation process (Brender & Zhang, 2015). Mutations in ND1 have been previously implicated in influencing metabolic efficiency in varied species including salmons (Consuegra, John, Verspoor, & Leaniz, 2015) and African elephants (Finch, Zhao, Korkin, Frederick, & Eggert, 2014). Finch et al., (2014) argue that mutations in ND1, ND4, ND5, ND6, and ATP6 genes in the African elephants are an indicative of adaptive evolution. Considering that mutations in ND1 detected in this study involved residues found at protein–protein binding sites where they interacted with residues in other subunits (Table 4), this may be a reflection of their potential importance in protein function, most likely in metabolic processes.

Other mutations detected in the current study were at residues in ND2, ND4, ND5, and ND6 as shown in Table 2. In mammalian mitogenome, ND2, ND4, and ND5 genes are regarded as the real proton pumps related to Na^+^/H^+^ antiporters of the Mrp family in OXPHOS complex I (Fonseca et al., 2008). These three subunits are connected by the arm of ND5, which allows for well‐coordinated shifts in proton pumping that consequently drive ATP production (Hunte, Zickermann, & Brandt, 2010). Changes (e.g., biochemical changes) in these subunits may hinder or improve proton‐pumping process (Fonseca et al., 2008). Mutations at ND4_87 _(found in all lineages except Clade 3), ND5_16_, ND5_186 _(found in all lineages except Clade 2/A), ND5_191 _(private to Clade 1 haplogroup C, D and E), and ND6_4_ (private to clade except for one sample) were all located in protein–protein interacting sites or/and translocation channels where they were likely to influence biochemical processes (Table 4). Interestingly, mutations in some of the aforementioned residues coincided with those reported elsewhere; ND5_16_ mutation is suggested to have a role in functional metabolic differences in *Tachycineta* swallow species inhabiting environments with latitudinal variations (Stager et al., 2014). Mutations at site ND5_16_ (of amphibians and coelacanths) and ND5_20 _(of amphibians, lungfishes, and coelacanths) are suggested to be linked to evolutionary adaptation to high atmospheric oxygen during cladogenesis (George & Blieck, 2011). Finch et al. (2014) in their study on African savanna and forest elephants also proposed that positive selection in complex I residues including ND5_20 _is indicative of ecological adaptations. With respect to site ND6_4_, mutations in residues adjacent to this site have been linked to adaptive selection, where amino acid replacements in ND6_6_ is suggested to influence efficiency of electron transfer under cold and hypoxic conditions in cephalopods (Almeida et al., 2015), while in rodents, ND6_5_ and ND6_6_ mutations are linked with adaptation to hypoxic conditions (Tomasco & Lessa, 2011). This evidence points to adaptive variations to oxygen availability and metabolic processes, which may be associated with possible metabolic adaptation of woolly mammoths clades in cold high‐latitude environments that are characterized with high oxygen pressure. This hypothesis may also be correlated with previous findings on the adaptation of woolly mammoth hemoglobin in maintaining stable O_2_ affinity in extremely cold environments (Campbell et al., 2010).

In our analyses, significant mutations were also detected in cyt *b*, ATP6, and COX1. Cyt *b* is involved in catalyses of the reversible transfer of electrons from ubiquinol to the cytochrome c coupled with proton translocation against the proton gradient (Q‐cycle). This proton gradient is utilized by ATP synthase to convert adenosine diphosphate (ADP) into adenosine triphosphate (ATP) (Saraste, 1999). Based on our biochemical analyses, we did not find clear evidence for functional relevancy of the mutations in cyt *b*, ATP6, and COX1, and their involvement in adaptive selection in woolly mammoths. However, we have taken note of previous findings where mutations in these sites were reported. In Gilbert, Drautz (Gilbert et al., 2008) study on woolly mammoths, mutations at cyt *b*_371 _and COX1_190_ were not suggested to have functional importance in metabolic processes. On the other hand, mutations at cyt *b*_300_ (in amphibians and lungfishes) and ATP6_110_ (in amphibians and coelacanths) have been linked with adaptation to increased oxygen levels (George & Blieck, 2011). Taking into consideration of a combination of this evidence with our results, where mutations in cyt *b*_300_ (shared by clade 1 and 2/A) and ATP6_110_ (private to clade 1) were only present in lineages that predominantly existed in persistently cold and dry environments in Northern Siberia and North America (Lister, Sher, Essen, & Wei, 2005; Muhs, Ager, Been, Bradbury, & Dean, 2003; Szpak et al., 2010), this raises a possibility that these substitutions might have contributed to a regulatory‐driven adaptation.

Given the expanded mitogenome dataset and robust inference methods employed in this study, we are confident that this work evaluated positive selection signals in mitochondrial protein‐coding genes across woolly mammoth's phylogeny. In conclusion, our findings provide compelling evidence for positive selection in eleven promising candidate residues in the OXPHOS genes, which suggests that these mutations might have played a role in metabolic adaptations among the lineages to their different environments. However, these inferences can be further tested in the future to provide more insights into the effects of these mutations in other systems and link their relevancy to shaping woolly mammoth's mitochondrial evolution.

## CONFLICT OF INTEREST

The authors declare no conflict of interest.

## AUTHOR'S CONTRIBUTIONS

JNN and YCX designed the study. TML prepared the complete mitochondrial genome sequences from whole genome sequences. JNN and TDD performed analyses, and JNN drafted the manuscript. LZ and AKA provided valuable suggestions during data analyses. All authors contributed to and approved the final manuscript.

## Data Availability

Mitochondrial genome sequences generated in this study have been stored in China National GeneBank (CNGB) Nucleotide Sequence Archive (CNSA: https://db.cngb.org/cnsa; Accession No. CNP0000277).
